# Screening for infectious diseases among newly arrived asylum seekers, Bavaria, Germany, 2015

**DOI:** 10.2807/1560-7917.ES.2018.23.10.17-00176

**Published:** 2018-03-08

**Authors:** Nikolaus Ackermann, Durdica Marosevic, Stefan Hörmansdorfer, Ute Eberle, Gabriele Rieder, Bianca Treis, Anja Berger, Heribert Bischoff, Katja Bengs, Regina Konrad, Wolfgang Hautmann, Katharina Schönberger, Anne Belting, Gisela Schlenk, Gabriele Margos, Martin Hoch, Friedrich Pürner, Volker Fingerle, Bernhard Liebl, Andreas Sing

**Affiliations:** 1 LGL Bayerisches Landesamt für Gesundheit und Lebensmittelsicherheit Oberschleißheim, Oberschleißheim, Germany; 2 These authors contributed equally to the paper; 3 European Public Health Microbiology Training Programme (EUPHEM), European Centre for Disease Prevention and Control (ECDC), Stockholm, Sweden

**Keywords:** asylum seeker health, tuberculosis, HIV, hepatitis B, louse-borne relapsing fever

## Abstract

As a consequence of socioeconomic and political crises in many parts of the world, many European Union/European Economic Area (EU/EEA) countries have faced an increasing number of migrants. In the German federal state of Bavaria, a mandatory health screening approach is implemented, where individuals applying for asylum have to undergo a medical examination that includes serological testing for HIV and hepatitis B, screening for tuberculosis, and until September 2015, stool examination for *Salmonella* spp. and *Shigella* spp.. **Methods**: Data from mandatory screening of all first-time asylum seekers in Bavaria in 2015 was extracted from the mandatory notification and laboratory information system and evaluated. **Results**: The HIV positivity and hepatitis B surface antigen (HBsAg) positivity rate of tested samples from asylum seekers were 0.3% and 3.3%, respectively, while detection rate of active tuberculosis was between 0.22% and 0.38%. The rates for HIV, hepatitis B, and tuberculosis among asylum seekers were similar to the corresponding prevalence rates in most of their respective countries of birth. Only 47 *Salmonella* spp. (0.1%) were isolated from stool samples: 45 enteric and two typhoid serovars. Beyond mandatory screening, louse-borne relapsing fever was found in 40 individuals. **Conclusions**: These results show that mandatory screening during 2015 in Bavaria yielded overall low positivity rates for all tested infectious diseases in asylum seekers. A focus of mandatory screening on specific diseases in asylum seekers originating from countries with higher prevalence of those diseases could facilitate early diagnosis and provision of treatment to affected individuals while saving resources.

## Introduction

Since the beginning of 2010, many European Union/European Economic Area (EU/EEA) countries have faced an increasing number of asylum seekers as a consequence of socioeconomic and political crises including war and civil unrest in many parts of the world. In 2015 in Germany, a considerable increase of registered asylum seekers was recorded [1]. The majority of asylum seekers tend to be in relatively good health such that the risk of an outbreak of infectious diseases in host countries is low [2-4]. Nevertheless, an established entry screening system for the presence of infectious diseases allows for early diagnosis and timely treatment for the affected individual, and a broad consensus therefore exists among many EU/EEA countries regarding the necessity of screening migrant populations, with considerable variations in the actual screening practices [1,5,6]

In Germany, according to the Federal Asylum Procedure Act (AsylG), asylum seekers are required to stay in a reception centre or in collective accommodation and have to undergo a mandatory medical examination for communicable diseases, including an X-ray of the respiratory organs [7]. The respective health authorities of the 16 German federal states determine the extent of the medical examination. In the federal state of Bavaria, this medical examination has to be performed within the first 3 days of arrival and consists of: (i) a physical examination to assess general health status and to screen for evident symptoms of an infectious disease; (ii) screening for active open pulmonary tuberculosis (TB); (iii) serological screening for an underlying HIV infection or hepatitis B in persons older than 15 years of age; (iv) a bacteriological stool examination for the presence of *Salmonella* spp. and *Shigella spp.;* and (v) a parasitological stool examination for the presence of ova and parasites in asylum seekers originating from countries with a high incidence of intestinal parasitic diseases, especially countries outside Europe and the Middle East.

This article reports the results of the mandatory screening procedures for HIV infection, hepatitis B, TB, salmonellosis and shigellosis, as well as the occurrence of louse-borne relapsing fever in asylum seekers arriving in Bavaria during 2015. It thus provides information on the health status of asylum seekers that arrived in Bavaria that year, and identifies disease-specific, high-risk countries of origin.

## Methods

### Demographic data

Information about age, sex and country of origin were recorded during each asylum seeker’s initial interview for registration with the Bavarian local health authorities. In this context, the reported country of origin is considered the birth country stated by the asylum seeker or recorded from their documents.

### Mandatory screening for tuberculosis and the notification system

Screening for active TB is mandatory for asylum seekers according to the AsylG, and is performed at the level of local health authorities. It involves a chest X-ray for those older than 15 years of age or an immunological assay, i.e. the tuberculin skin test (TST) or interferon gamma release assay (IGRA) for those between 10 and 15 years of age or persons in whom an X-ray is contraindicated. Respiratory material from asylum seekers with a suspicious X-ray or positive TST or IGRA was subjected to further testing by microscopy, PCR or culture to elaborate on the infectivity of the disease. In this report, infectious TB was considered if respiratory material was available and tested positive for *Mycobacterium tuberculosis* complex via microscopy, PCR or culture.

All positive results from TB screening, i.e. all cases of active TB in asylum seekers either clinically and/or diagnostically detected, were entered into the mandatory notification system for all active TB cases in Germany, together with demographics of the asylum seeker, including country of origin. The information on active TB cases from the mandatory TB screening of asylum seekers from 2011 to 2015 was extracted from the mandatory notification system. However, because the number of screened asylum seekers that were negative for active TB in Bavaria was not collected as part of the German Infection Protection Act (IfSG) notification data, an exact active TB positivity rate in asylum seekers detected by the mandatory TB screening based on an exact denominator per year and per country of origin could not be calculated. Thus, to estimate the positivity rate of active TB in asylum seekers in 2015, the following two numbers were used as an approximate denominator: (i) the known number of samples per country of origin received for serological screening in 2015 in Bavaria (n = 95,117 tested HIV samples from asylum seekers) and (ii) the estimated number of allocated asylum seekers to Bavaria in 2015 (n = 169,448, ca 15.52% of 1,091,894 asylum seekers registered in Germany via the EASY registration system and the Königstein distribution formula) [1,8]. The data for the years 2011 to 2014 are presented as absolute numbers to give a mean of comparison and context for the interpretation of the data presented for 2015.

### Mandatory screening for HIV, hepatitis B, salmonellosis and shigellosis

Screening for HIV and hepatitis B by blood sampling is mandatory for asylum seekers older than 15 years of age according to AsylG. Based on Bavarian administrative regulations, laboratory procedures on samples from asylum seekers were performed at the Bavarian Health and Food Safety Authority (LGL). All information about demographics, country of origin and negatively screened tests was available and extracted from the laboratory database. Therefore, overall positivity rates and positivity rates by country of origin were calculated.

Serum samples of asylum seekers were screened for HIV infection with the Abbott ARCHITECT HIV Ag/Ab Combo (Abbott Diagnostics, Wiesbaden, Germany) according to the manufacturer’s instructions. Samples reactive in the screening test underwent two confirmatory tests: (i) the INNO-LIA HIV I/II Score (Fujirebio, Gent, Belgium), and (ii) the Abbott RealTi*m*e HIV-1 (Abbott, Illinois, United States (US)). Hepatitis B virus screening included surface antigen (HBsAg) detection performed with the Abbott ARCHITECT HBsAg Qualitative II assay (Abbott, Illinois, US). All Abbott ARCHITECT assays are chemiluminescent magnetic microparticle-based immunoassays run on the automated random access instrument i2000SRe.

At the LGL, mandatory bacteriological screening of stool samples from all asylum seekers was performed until September 2015, with a symptom-based stool sampling policy implemented thereafter. Therefore, all information about the demographics, country of origin and negatively screened tests was available and extracted from the laboratory database. Stool samples for bacteriological screening were suspended in a ratio of 1:10 in selenite broth and cultured onto XLD-Agar and CHROMagar Salmonella Plus. After 24 hours the enriched selenite broth was sub-cultured on BPLS agar, suspicious colonies were sub-cultured on triple sugar iron-agar (Kligler), Endo agar, and XLD agar. *Shigella* spp. were differentiated biochemically by standard methods while *Salmonella* spp. were identified by MALDI-TOF Mass Spectrometry (Bruker, Bremen, Germany). Both species were also differentiated serologically.

### Diagnosis of louse-borne relapsing fever

Screening for louse-borne relapsing fever (LBRF) is not mandatory according to AsylG, but was diagnosed clinically in some asylum seekers because of its characteristic symptoms and confirmed by microscopy and serology [9].

Slide microscopy was performed after standard Giemsa staining of blood films, DNA extraction and PCR targeting 16S rRNA [10], *flaB* [11] and *glp*Q [12] followed by sequencing was done from 1 ml ethylenediaminetetraacetic acid (EDTA)-blood as described previously [9].

Only absolute numbers of symptomatic and diagnosed patients are reported here.

## Results

### Tuberculosis

In 2015, a total of 365 active TB cases among asylum seekers were notified in Bavaria via the regular IfSG notification system [13]. Compared with 2014, this represented a threefold increase of TB cases (Table 1). However, the proportion of infectious active TB in asylum seekers did not change in comparison to the previous years 2013 and 2014; 42.7% were infectious and 46.3% were diagnosed as non-infectious TB cases in 2015. For the remaining 11.0% of the notified TB cases, no additional information on direct pathogen detection was available. Of the cases, 79.6% were aged between 15 and 34 years. The positivity rate of cases of active TB at entry screening for asylum seekers was estimated to be in the order of 0.22% and 0.38% using as denominators, the number of asylum seekers allocated to Bavaria via the federal distribution system (n = 169,448) and the number of serologically screened asylum seekers in Bavaria (n = 95,117), respectively (Table 2).

**Table 1:** Overview of active tuberculosis cases in asylum seekers based on IfSG notification data^a^, Bavaria, Germany, 2011–2015

Characteristic		Year of first diagnosis during screening investigation										Total (n = 578)
		2011 (n = 14)		2012 (n = 21)		2013 (n = 59)		2014 (n = 119)		2015 (n = 365)		
		n	%	n	%	n	%	n	%	n	%	
Sex	Male	11	78.6	15	71.4	44	74.6	105	88.2	333	90.7	508
	Female	3	21.4	6	28.6	15	25.4	14	11.8	28	7.6	66
	Unknown	0	0.0	0	0.0	0	0.0	0	0.0	4	1.7	4
Age	0–14 years	2	14.3	0	0.0	2	3.4	3	2.5	2	0.5	9
	15–24 years	3	21.4	10	47.6	13	22.0	65	54.6	189	51.5	280
	25–34 years	7	50.0	6	28.6	32	54.2	28	23.5	103	28.1	176
	35–49 years	1	7.1	2	9.5	4	6.8	18	15.1	57	15.5	82
	≥ 50 years	1	7.1	3	14.3	8	13.6	5	4.2	14	3.8	31
	unknown	0	0.0	0	0.0	0	0.0	0	0.0	0	0.0	0
Resistance	MDR	0	0.0	0	0.0	4	6.8	1	0.8	9	2.5	14
	XDR	0	0.0	0	0.0	0	0.0	0	0.0	2	0.5	2
Site of disease	Lung	12	85.7	17	81.0	57	96.6	106	89.1	328	89.4	520
	Lymph node (extra-thoracic)	1	7.1	0	0.0	0	0.0	3	2.5	8	2.2	12
	Lymph node (intra-thoracic)	1	7.1	0	0.0	0	0.0	5	4.2	11	3.0	17
	Pleura	0	0.0	0	0.0	1	1.7	3	2.5	12	3.3	16
	Meninges	0	0.0	1	4.8	0	0.0	0	0.0	0	0.0	1
	Urogenital tract	0	0.0	0	0.0	0	0.0	1	0.8	0	0.0	1
	Spine	0	0.0	1	4.8	0	0.0	1	0.8	2	0.5	4
	Other bones and joints	0	0.0	1	4.8	0	0.0	0	0.0	3	0.8	4
	Other/unknown	0	0.0	1	4.8	1	1.7	0	0.0	1	0.3	3
Direct pathogen detection^b^	Yes	8	57.1	4	19.0	28	47.5	53	44.5	156	42.7	249
	No	5	35.7	16	76.2	29	49.2	54	45.4	169	46.3	273
	Unknown	1	7.2	1	4.8	2	3.3	12	10.1	40	11.0	56

IfSG: German Infection Protection Act; MDR: multidrug-resistant; TB: tuberculosis; XDR: extensively drug-resistant.

^a^ Data status: 13 June 2016 [13].

**^b^** Direct pathogen detection includes a positive result of either PCR, microscopy or culture from respiratory material.

**Table 2:** Countries of origin of notified active tuberculosis cases in asylum seekers based on IfSG notification data^a^, Bavaria, Germany, 2011–2015

Country of origin	2011 (n)	2012 (n)	2013 (n)	2014 (n)	2015 (n)	2015 active TB cases (%)^b^	TB incidence rate/100,000 population (95% CI) according to WHO^d^
**Eastern Africa**							
Eritrea	0	0	0	18	47	1.16	65 (30–113)
Ethiopia^d^	1	2	1	1	22	1.18	192 (142–250)
Somalia	4	3	2	38	65	2.71	274 (177–391)
**Middle Africa**							
Democratic Republic of the Congo^d^	0	1	2	0	3	2.73	324 (210–463)
Gabon	0	0	0	0	1	NA	465 (344–604)
**Western Africa**							
Mali	0	0	0	3	8	1.15	57 (37–81)
Nigeria^d^	0	1	2	3	19	0.52	322 (189–488)
Senegal	0	0	4	12	15	0.84	139 (90–198)
Sierra Leone^d^	0	0	1	3	11	2.50	307 (198–438)
**South-central Asia**							
Afghanistan	2	4	7	9	48	0.30	189 (122–270)
Pakistan^d^	0	0	6	3	36	0.80	270 (175–386)
**Western Asia**							
Armenia	0	0	2	0	2	NA	41 (36–46)
Azerbaijan	3	0	0	1	9	NA	69 (57–83)
Georgia	0	0	5	3	5	1.39	99 (80–120)
Iraq	2	2	2	0	1	0.01	43 (38–49)
Syria	1	0	2	5	26	0.09	20 (15–25)
**Eastern Europe**							
Ukraine	0	0	0	1	8	0.35	91 (59–130)
**Southern Europe**							
Albania	0	1	1	0	3	0.07	19 (16–22)
Bosnia and Herzegovina	0	0	1	2	2	NA	37 (29–47)
Kosovo*	0	0	0	1	12	NA	NA
Serbia	0	2	0	2	2	NA	21 (19–24)
**Other countries**	**1**	**5**	**21**	**14**	**20**	**NA**	**75 (58–94)**
**Total**	**14**	**21**	**59**	**119**	**365**	**0.22–0.38^c^**	**–**

CI: confidence interval; IfSG: German Infection Protection Act; NA: not available; TB: tuberculosis; WHO: World Health Organization.

^a^ Data status: 13 June 2016 [13].

^b^ An approximate value estimated using the number of samples from asylum seekers tested for HIV in Bavaria in 2015 for each country of origin as the denominator (see Table 5), as the number of performed X-ray screenings is unknown.

^c^ The total percentage of active TB cases was calculated using as denominators, the number of asylum seekers registered through the EASY registration system in 2015 in Bavaria and with the Königstein distribution formula applied (n = 169,448) and the number of tested samples for HIV (n = 95,117, see Table 5).

^d^ High burden TB countries according to the WHO [14].

*This designation is without prejudice to positions on status, and is in line with United Nations Security Council Resolution 1244/99 and the International Court of Justice Opinion on the Kosovo declaration of independence.

Among the most often reported countries of origin from patients diagnosed with TB were countries from Western (Nigeria, Senegal, Sierra Leone) and Eastern Africa (Eritrea, Ethiopia, Somalia) as well as from South-central Asia (Afghanistan, Pakistan) (Table 2). Four of them belong to the 30 high burden TB countries as defined by the World Health Organization (WHO) global TB report: Pakistan (n = 36), Ethiopia (n = 22), Nigeria (n = 19), and Sierra Leone (n = 11) [14]. However, the highest number of asylum seekers diagnosed with TB in Bavaria in 2015 came from Somalia (n = 65) and Afghanistan (n = 48). Twenty-six TB cases were detected among asylum seekers from Syria, which was the fifth most common country of origin among asylum seekers diagnosed with TB in Bavaria in 2015.

Of all TB infections, 89.4% affected the lung, but extrapulmonary TB cases (e.g. in lymph nodes (5.2 %) or bones (1.3 %)) were also diagnosed.

Nine imported multidrug-resistant (MDR) TB cases were notified in 2015, as compared with one case in 2014 and four in 2013 (Table 1). Cases notified in 2015 originated from Afghanistan, Azerbaijan, Eritrea, Gabon, Georgia, Russia, Somalia and Ukraine. For one patient, information about the country of origin was missing. During the last 5 years, only two cases of extensively drug-resistant (XDR) TB were diagnosed, both in 2015, with countries of origin of Russia and Azerbaijan (Table 1). Although there was an observed increase in total numbers of TB and MDR/XDR TB cases among asylum seekers from 2011 to 2015, the difference in the proportion of MDR/XDR TB cases among TB cases in asylum seekers arriving in Bavaria was not statistically significant (p > 0.05) compared with the proportion of MDR/XDR TB cases among TB cases in the non-asylum population (Table 3).

**Table 3:** Comparison of multidrug-resistant/extensively drug-resistant and non-resistant tuberculosis cases in non-asylum seekers and asylum seekers based on IfSG notification data^a^, Bavaria, Germany, 2011–2015

Year	Non-asylum seekers			Asylum seekers			p value
	TB (n)	MDR/XDR TB (n)	Proportion (%, 95% CI)	TB (n)	MDR/XDR TB (n)	Proportion (%, 95% CI)	
2011	661	7	1.06 (0.1–2.2)	14	0	0.00	0.70
2012	635	12	1.89 (1.0–3.2)	21	0	0.00	0.53
2013	523	11	2.10 (1.1–3.7)	59	4	6.78 (1.9–16.5)	0.09
2014	573	12	2.09 (1.1–3.6)	119	1	0.84 (0.02–4.6)	0.59
2015	695	16	2.30 (1.3–3.7)	365	11	3.01 (1.5–5.3)	0.62

CI: confidence interval; IfSG: German Infection Protection Act; MDR: multidrug-resistant; TB: tuberculosis; XDR: extensively drug-resistant.

^a^ Data status: 13 June 2016 [13].

### HIV and hepatitis B

During 2015, a total of 95,117 and 94,843 serum samples from asylum seekers were tested for HIV and hepatitis B at the Bavarian LGL, respectively. This represented a threefold increase compared with 2014 (27,103, table not shown). In 2015, 58.1% of the HIV samples were from males and 15.9% from females; 26.0% did not have any information about sex. The highest proportion of samples, 24.5%, was from individuals 15 to 24 years of age. However, in almost half of all cases (46.1%), age was not reported (Table 4). Among males, the highest proportion of samples was from the 15 to 24 age group (35.2%), while among females, the highest proportion was from those aged 24 to 35 years (26.0%) (Table 4).

**Table 4:** Age and sex distribution of asylum seekers with samples examined for HIV, Bavaria, Germany, 2015 (n = 95,117)

Age		Female		Male		Unknown		Total
		n	%^a^	n	%^a^	n	%^a^	
0–14 years	n	172	30.9	381	68.4	4	0.7	557
	%^b^	1.1	–	0.7	–	0.0	–	0.6
15–24 years	n	3,535	15.2	19,457	83.6	292	1.2	23,284
	%^b^	23.4	–	35.2	–	1.2	–	24.5
25–34 years	n	3,915	24.0	12,213	74.8	190	1.2	16,318
	%^b^	26.0	–	22.1	–	0.8	–	17.2
35–49 years	n	2,532	28.8	6,164	70.0	105	1.2	8,801
	%^b^	16.8	–	11.1	–	0.4	–	9.3
≥ 50 years	n	788	35.9	1,380	62.9	26	1.2	2,194
	%^b^	5.2	–	2.5	–	0.1	–	2.3
Unknown	n	4,139	9.4	15,718	35.8	24106	54.8	43,963
	%^b^	27.5	–	28.4	–	97.5	–	46.1
**Total**	**n**	**15,081**	**15.9**	**55,313**	**58.1**	**24722**	**26.0**	**95,117**

^a^ Percentage of males and females by age group.

^b^ Percentage of age groups by sex.

Most samples for HIV and hepatitis B testing were from asylum seekers from Syria (n = 30,450 and 30,415), Afghanistan (n = 16,227 and 16,176) and Iraq (n = 8,185 and 8,178), respectively (Tables 5 and Table 6).

**Table 5:** Number of samples tested for HIV, screening test reactivity and confirmation test positivity rate^a^, Bavaria, Germany, 2015 (n = 95,117)

Country of origin	Number of tested samples	HIV screening test reactivity rate (%)	HIV positivity rate (%, 95% CI)	Prevalence of HIV among adults 15–49 years of age according to WHO^b^ (%, 95% CI)
**Eastern Africa**				
Eritrea	4,068	1.7	1.2 (0.9–1.6)	0.7 (0.5–1.0)
Ethiopia	1,869	1.6	1.4 (0.9–2.0)	1.2 (1.0–1.5)
Somalia	2,396	0.9	0.5 (0.3–0.9)	0.5 (0.4–0.7)
Uganda	139	4.3	4.3 (1.6–9.2)	7.3 (6.6–8.1)
**Middle Africa**				
Congo	110	6.4	6.4 (2.6–12.7)	2.8 (2.5–3.0)
Democratic Republic of the Congo	63	12.7	11.1 (4.6–21.6)	1.0 (0.9–1.2)
**Western Africa**				
Mali	695	2.0	0.9 (0.3–1.9)	1.4 (1.2–1.7)
Nigeria	3,629	3.6	2.3 (1.9–2.9)	3.2 (2.9–3.4)
Senegal	1,789	2.0	1.0 (0.6–1.5)	0.5 (0.4–0.6)
Sierra Leone	440	4.1	3.0 (1.6–5.0)	1.4 (1.2–1.6)
**South-central Asia**				
Afghanistan	16,227	0.3	0.05 (0.021–0.09)	< 0.1 (< 0.1–< 0.1)
Iran	1,940	0.7	0.4 (0.18–0.8)	0.1 (0.1–0.2)
Pakistan	4,502	0.4	0.2 (0.06–0.32)	0.1 (0.1–0.2)
**Western Asia**				
Georgia	360	0.6	0.6 (0.07–2.0)	0.3 (0.2–0.4)
Iraq	8,185	0.2	0.02 (0.003–0.08)	NA
Syria	30,450	0.3	0.03 (0.016–0.06)	< 0.1 (< 0.1–< 0.1)
**Eastern Europe**				
Russia	293	1.4	1.4 (0.4–3.5)	0.5 (0.4–0.6)
Ukraine	2,303	1.4	1.2 (0.8–1.7)	NA
**Southern Europe**				
Albania	4,479	0.4	0.1 (0.04–0.26)	NA
**Other countries**	**11,180**	**0.4**	**0.2 (0.1–0.3)**	**–**
**Total**	**95,117**	**0.7**	**0.3 (0.25–0.32)**	–

CI: confidence interval; NA: not available; WHO: World Health Organization.

^a^ All laboratory testing occurred at the Bavarian Health and Food Safety Authority (LGL).

^b^ Prevalence of HIV among adults 15–49 years of age in 2014 by most common reported countries of birth according to the WHO [23].

**Table 6:** Number of tested samples, HBsAg-positive serum samples^a^, and the calculated HBsAg positivity rate, Bavaria, Germany, 2015 (n = 94,843)

Country of origin	Number of tested samples	Number of HBsAg positive samples	HBsAg positivity rate (%, 95% CI)	HBsAg prevalence according to Schweitzer et al.^b^ (%, 95% CI)
**Eastern Africa**				
Eritrea	4,034	199	4.9 (4.3–5.6)	2.5 (2.3–2.7)
Ethiopia	1,856	133	7.2 (6.0–8.4)	6 (5.8–6.3)
Somalia	2,389	105	4.4 (3.6–5.2)	14.8 (13.8–15.8)
Uganda	136	4	2.9 (0.8–7.4)	9.2 (8.7–9.8)
**Middle Africa**				
Congo	107	9	8.4 (3.9–15.4)	11 (9.8–12.3)
Democratic Republic of the Congo	61	7	11.5 (4.7–22.2)	6 (5.7–6.3)
**Western Africa**				
The Gambia	123	4	3.3 (0.9–8.1)	12.3 (11.5–13.1)
Ghana	47	12	25.5 (13.9–40.3)	12.9 (12.4–13.4)
Mali	693	107	15.4 (12.8–18.3)	13.1 (12.7–13.5)
Niger	52	6	11.5 (4.4–23.4)	15.5 (14.4–16.7)
Nigeria	3,602	166	4.6 (3.9–5.3)	9.8 (9.6–9.9)
Senegal	1,787	289	16.2 (14.5–17.9)	11.1 (10.7–11.4)
Sierra Leone	438	77	17.6 (14.1–21.5)	8.4 (5.99–11.73)
**South-central Asia**				
Afghanistan	16,176	633	3.9 (3.6–4.2)	1.6 (1.3–2.0)
Iran	1,933	18	0.9 (0.5–1.4)	0.96 (0.95–0.96)
Pakistan	4,501	156	3.5 (2.9–4.0)	2.8 (2.7–2.8)
**South-eastern Asia**				
Vietnam	74	5	6.8 (2.2–15.0)	10.8 (10.3–11.3)
**Western Asia**				
Armenia	316	10	3.2 (1.5–5.7)	NA
Azerbaijan	442	25	5.7 (3.7–8.2)	2.8 (1.7–4.5)
Georgia	359	18	5.0 (3.0–7.8)	2.6 (2.2–3.1)
Iraq	8,178	78	1.0 (0.7–1.1)	0.7 (0.65–0.70)
Syria	30,415	507	1.7 (1.5–1.8)	2.6 (2.2–3.2)
Turkey	113	5	4.4 (1.4–10.0)	4.0 (3.99–4.02)
**Eastern Europe**				
Belarus	198	4	2.0 (0.5–5.1)	4.6 (4.2–5.0)
Russia	296	11	3.7 (1.9–6.6)	2.73 (2.6–2.8)
Ukraine	2,295	48	2.1 (1.5–2.7)	1.5 (1.1–1.9)
**Southern Europe**				
Albania	4,481	287	6.4 (5.7–7.1)	7.8 (7.6–8.0)
Bosnia and Herzegovina	397	9	2.3 (1.0–4.3)	1.1 (0.9–1.4)
Kosovo*	4,769	106	2.2 (1.8–2.6)	4.2 (4.0–4.3)
Serbia	688	14	2.0 (1.1–3.4)	0.5 (0.4–0.6)
The former Yugoslav Republic of Macedonia	435	25	5.7 (3.8–8.4)	NA
**Other countries**	**3,452**	**98**	**2.8 (2.3–3.4)**	**–**
**Total**	**94,843**	**3175**	**3.3 (3.2–3.5)**	**–**

CI: confidence interval; HBsAg: hepatitis B virus screening included surface antigen; NA: not available.

^a^ All laboratory testing occurred at the Bavarian Health and Food Safety Authority (LGL).

^b^ National HBsAg prevalence as reported by Schweitzer et al. [24].

*This designation is without prejudice to positions on status, and is in line with United Nations Security Council Resolution 1244/99 and the International Court of Justice Opinion on the Kosovo declaration of independence.

In 2015, 0.7% of 95,117 serum samples from asylum seekers screened for HIV yielded a reactive test result (Table 5). Of the reactive screening tests, 0.3% were positive for HIV using confirmatory HIV blot and HIV PCR tests. Of these HIV positive samples, 71.4% (227 of 318) were from individuals originating from sub-Saharan African countries. Confirmatory tests for reactive HIV screening tests were more often negative from people originating from low prevalence countries, such as Syria or Afghanistan, while a higher confirmation rate was found for screening tests from high prevalence countries, such as Nigeria, Democratic Republic of the Congo or Congo.

Serological hepatitis B testing was performed on 94,843 serum samples (Table 6). HBsAg, an indicator of hepatitis B virus infection and likely infectiousness, was detected in 3.3% of all tested samples. The highest HBsAg positivity rates were found in asylum seekers from Sierra Leone, Senegal and Mali, with positivity rates of 17.6%, 16.2% and 15.4%, respectively. HBsAg positivity rates in people from the main countries of origin in 2015, Syria, Afghanistan and Iraq, were 1.7%, 3.9% and 1.0%, respectively.

### Salmonellosis and shigellosis

A total of 53,085 stool samples were examined bacteriologically in 2015 with only very low detection rates for enteropathogenic bacteria: 47 *Salmonella* spp. (0.1%) were grown, 45 enteric serovars (0.1%) and two typhoid serovars (0.004%). The latter two were *Salmonella* Typhi from an asylum seeker from Nigeria and *S.* Paratyphi B from an asylum seeker from Albania. The most frequently isolated serovars were *S.* Enteritidis (n = 10), *S.* Typhimurium (n = 7) and *S.* Kentucky (n = 3). Only five *Shigella* spp. (0.01%) were isolated, four isolates of *Shigella flexneri* from asylum seekers from Somalia, Eritrea, Ethiopia and an unknown country, and one isolate of *Shigella sonnei* from an asylum seeker from Syria.

### Louse-borne relapsing fever

Beyond the mandatory entrance screening in Bavaria, a total of 40 cases of LBRF caused by *Borrelia recurrentis* were confirmed in asylum seekers in Bavaria. These 40 cases include the case series of 15 individuals reported on by Hoch et al. [9]. Notably, for 11 individuals, diagnosis of LBRF was possible by PCR and sequencing only, while microscopy was negative. LBRF was almost exclusively found in males (n = 38/40). Ages of all 40 cases ranged from 14 to 36 years. The majority of cases (n = 37) originated from Somalia, while two were from Eritrea and one was from Ethiopia. In 14 cases, information about the onset of symptoms and/or the arrival of the cases in Bavaria were not available. Fifteen cases reported an onset of symptoms before their arrival in Bavaria, seven cases reported an onset of symptoms within the first 6 days after arrival and four cases were diagnosed immediately upon arrival as their clinical symptoms were severe and they did not report having symptoms before.

## Discussion

### Tuberculosis

During 2011 to 2014, the number of all notified TB cases in Bavaria was stable ranging from 582 to 692 cases per year, including TB cases among asylum seekers. In 2015, the number of all notified TB cases increased to 1,060, of which a third (n = 365) were in asylum seekers diagnosed during mandatory screening, while in previous years they contributed on average 8% (Table 3). This observed increase in notified TB cases in 2015 was not unexpected, as there was an approximately threefold increase in the number of screened people in 2015 compared with 2014 in Bavaria. This corresponded in 2015 to a TB positivity rate among asylum seekers of 0.22% to 0.38%, which was comparable to a range described in a systematic literature review from 2009 that assessed the effectiveness of TB screening methods and strategies in migrants in EU and EEA countries. This review found a median yield of TB of 0.18% (interquartile range: 0.10–0.35) [15].

It is reasonable to suppose that active screening of asylum seekers is a more sensitive approach and will detect more TB cases than the passive case finding approach applied for the general population. The active screening approach might thus explain the relatively low proportion of screening-related TB cases confirmed by pathogen detection in asylum seekers (42.7%) compared with a higher proportion of microbiological confirmations of active TB cases in the German population (ca 80%) [16]. The low confirmatory rate in this study is in accordance with other studies where only ca 40% of all screened immigrants with an X-ray suggestive of pulmonary TB were confirmed by direct pathogen detection [17-19]. This has important mutually opposing public health implications. For one, higher sensitivity by active screening will automatically result in lower specificity such that some individuals undergoing active screening with abnormal X-ray and without pathogen detection would be falsely diagnosed as TB cases and subjected to unnecessary long-term antibiotic treatment. On the other hand, active screening may allow early detection of TB, when specific symptoms have not yet developed, and thus provide early treatment options [18-20]. Furthermore, pulmonary TB cases identified through active screening in asylum seekers have been shown to have lower hospitalisation rates [19] and lower in-hospital mortality compared with residents [18,19].

No statistically significant difference was found between MDR/XDR-TB cases occurring among asylum seekers compared with MDR/XDR-TB cases in the general population (Table 3). This could be attributed to the countries of origin of asylum seekers arriving in Bavaria in 2015, as the majority originated from countries with low MDR-TB prevalence. Notable is the occurrence of a few MDR-TB cases from Azerbaijan, Somalia, Russia and Ukraine, all belonging to high MDR-TB burden countries. This is an important observation to take into account by public health officials and policy makers in countries receiving and accommodating asylum seekers from high MDR-TB burden countries [14].

### HIV

Of 95,117 serum samples from asylum seekers screened for HIV in 2015 in Bavaria, 0.7% yielded a reactive screening test result (Table 5). The screening test had a high sensitivity of 99.94%, but a specificity of 98.78% [21]; therefore, when applied to the high number of samples from people with a low pre-test probability, a considerable number of false positives was obtained. Not surprisingly, less than half of the reactive screening tests were positive in the confirmatory test, yielding a total of 0.3% of diagnosed HIV infections from the screened samples. The confirmatory tests were more often negative when performed for reactive screening tests from people originating from low prevalence countries, such as Syria or Afghanistan, the two main countries of origin. A total of 30,450 serum samples from people from Syria and 16,227 samples from people from Afghanistan were screened, together constituting 49% of all samples, but yielding only 0.03% and 0.05% positivity rate, respectively. For people originating from countries with higher HIV prevalence such as Nigeria and Uganda, a higher confirmation of the reactivity rate of the HIV screening test was detected. The HIV positivity rates of our tested samples from asylum seekers of different birth countries were in accordance with the corresponding HIV prevalence rates in their respective countries published by the Joint United Nations Programme on HIV/AIDS (UNAIDS) [22] and WHO [23], with only a few exceptions, e.g. Democratic Republic of the Congo. Considering the very low positivity rate among serum samples from people from Syria and Afghanistan, the invested time, money and effort in the diagnosis of new HIV cases among asylum seekers from these two countries is questionable and should be re-evaluated from a public health perspective. A mandatory screening approach only for HIV in asylum seekers originating from countries with higher prevalence of HIV, like the sub-Saharan African countries would have been sufficient to detect over 70% of HIV positive samples.

### Hepatitis B

Our HBsAg serum sample positivity rate of 3.3% (95% confidence interval (CI): 3.2–3.5) was not surprising considering that the majority of asylum seekers came from countries of the WHO Eastern Mediterranean Region (67% from Syria, Afghanistan, Iraq, Pakistan, Somalia), which is an area of lower intermediate hepatitis B endemicity (3.01%) according to a systematic review on hepatitis B prevalence that defined countries with lower intermediate hepatitis B endemicity as those with a hepatitis B prevalence rate between 2.00–4.99% [24]. This is in accordance with the results of a meta-analysis and of a longitudinal prospective study on seroprevalence of hepatitis B among immigrants and asylum seekers, where the prevalence among migrants mirrored the prevalence in their regions of origin [25,26]. Small discrepancies in the estimated country prevalence and our data could be due to the fact that the data by Schweitzer et al. [24] were extracted from available general population studies (blood donors, healthcare workers and pregnant women) without focus on asylum seeker or migrant populations. Furthermore, the differing numbers of participants in the systematic review of Schweitzer et al. and our data set could have influenced the prevalence (4,039 vs 30,415 for Syria, 4,511 vs 16,176 for Afghanistan and 495,998 vs 8,178 for Iraq) [24].

Sub-Saharan Africa is classified as a high endemicity area for hepatitis B [24,27]. In our study, the highest sample positivity was found in asylum seekers from Sierra Leone, Senegal and Mali (17.6%, 16.2%, and 15.4%, respectively). Interestingly, serum samples from people arriving from some countries like Somalia, Uganda, Nigeria and the Gambia had lower positivity rates compared with the prevalence estimated for their countries of origin. This might be due to the fact that studies included in the worldwide HBsAg seroprevalence estimates relied on available data, which often included healthcare worker data. This occupation in resource-poor countries is considered a risk factor for acquiring a hepatitis B infection and inclusion of these workers in seroprevalence studies might overestimate the country prevalence [28]. As serum samples from asylum seekers from almost every country of origin except for Iran (0.9%) and Iraq (1.0%) had a markedly higher HBsAg positivity rate in comparison to the endemicity rate in Germany (0.7%) [25], the mandatory hepatitis B screening approach for all asylum seekers seems justified. However, direct comparison of data between asylum seekers and the resident population in Germany is difficult because the inherently different methodology of data collection. Active surveillance by mandatory screening of a complete subpopulation vs passive surveillance via mandatory notification of persons tested only if the disease is suspected, often with clinical symptoms, have different sources and extents of bias. For instance, under-reporting and consequent underestimation might be expected to happen more often in a passive than in an active surveillance approach.

### Salmonellosis and shigellosis

Our data showed that *Salmonella* spp. and *Shigella* spp. are not widespread among asylum seekers arriving in Bavaria, particularly for typhoid serovars *S*. Typhi and *S*. Paratyphi A, B and C. Therefore, there are no indications that asylum seekers play a role as reservoirs for *Salmonella* spp. and *Shigella* spp. and they thus do not pose an infectious risk for the resident population in Germany. Moreover, the prevalence of *Salmonella* spp. and *Shigella* spp. among asylum seekers in Bavaria in 2015 were both within their respective 2011 to 2014 ranges for this group (data not shown). These persistently low prevalences led the Bavarian State Ministry of Health and Care to replace mandatory stool sampling with a symptom-based stool sampling policy from early September 2015 onwards.

### Louse-borne relapsing fever

A noteworthy disease occurring among a small number of asylum seekers during 2015 was LBRF, caused by *B. recurrentis*. This human-restricted pathogen transmitted by the body louse *Pediculus humanus humanus* is endemic in the Horn of Africa and has been the causative agent of large LBRF outbreaks in the past, usually associated with wars, civil unrest and extreme poverty [29,30]. Risk factors like low socioeconomic status, over-crowding and lacking personal hygiene facilities can promote the spread of the vector and disease, especially in refugee camps and shelters [30]. LBRF was a largely unknown disease in Germany until 2015 as only three cases had been notified since 1999, with these being in returning travellers [31]. However, a 2015 report on cases of LBRF in asylum seekers in Bavaria raised awareness about this re-emerging disease in medical and public health communities [9]. In this report, the main migration route could be narrowed down to the central Mediterranean route from Libya to Italy followed by a transit across Austria to Bavaria.

Almost all of the 40 LBRF-diagnoses were found in young male asylum seekers from Somalia, which could in part be explained by the age and sex distribution of asylum seekers arriving from there (data not shown). However, little information is known on the presence of LBRF in Somalia. Taking available information on the onset of symptoms, incubation periods, overlapping migration routes, and information obtained from whole genome sequencing of some of the strains into account [32], and in accordance with the findings of Hoch et al. [9], we hypothesise that infection may have taken place in Libya, during the journey across the Mediterranean Sea or Italy. In support of this is the fact that no timely delayed cases were detected which would have been indicative of a transmission in Germany.

In addition to some of the limitations mentioned earlier, e.g. the inability to tell where diseases were acquired and methodological issues in data collection, another limitation is that in this paper, ’country of origin’ is considered the birth country as stated by the asylum seeker or recorded from their documents.

## Conclusions

The prevalence of TB, HIV and hepatitis B in asylum seekers arriving in Bavaria was generally very consistent with the reported prevalence in the respective countries of origin. Overall, it can be concluded that for most notifiable infectious diseases, the low absolute number of infectious cases among asylum seekers screened in Bavaria, suggests no additional risk for the resident population. The main concern from a public health perspective might be the increase in absolute numbers of notified TB cases compared with the previous years. This highlights the importance of early TB case finding and consequent follow-up for both the asylum seeker population and the resident population. As a result of the low prevalence of *Salmonella* spp. and *Shigella* spp. found in the screening, a symptom-based screening was implemented in Bavaria.

A country-based screening approach appears to be reasonable and resource-effective, since our data are in accordance with the reported national HIV and hepatitis B prevalence data for different countries of origin. Mandatory screening of specific diseases in asylum seekers originating from countries with higher prevalence of those diseases could facilitate early diagnosis and therefore provision of treatment to affected individuals while saving resources.
